# ONRLDB—manually curated database of experimentally validated ligands for orphan nuclear receptors: insights into new drug discovery

**DOI:** 10.1093/database/bav112

**Published:** 2015-11-04

**Authors:** Ravikanth Nanduri, Isha Bhutani, Arun Kumar Somavarapu, Sahil Mahajan, Raman Parkesh, Pawan Gupta

## Abstract

Orphan nuclear receptors are potential therapeutic targets. The Orphan Nuclear Receptor Ligand Binding Database (ONRLDB) is an interactive, comprehensive and manually curated database of small molecule ligands targeting orphan nuclear receptors. Currently, ONRLDB consists of ∼11 000 ligands, of which ∼6500 are unique. All entries include information for the ligand, such as EC50 and IC50, number of aromatic rings and rotatable bonds, XlogP, hydrogen donor and acceptor count, molecular weight (MW) and structure. ONRLDB is a cross-platform database, where either the cognate small molecule modulators of a receptor or the cognate receptors to a ligand can be searched. The database can be searched using three methods: text search, advanced search or similarity search. Substructure search, cataloguing tools, and clustering tools can be used to perform advanced analysis of the ligand based on chemical similarity fingerprints, hierarchical clustering, binning partition and multidimensional scaling. These tools, together with the Tree function provided, deliver an interactive platform and a comprehensive resource for identification of common and unique scaffolds. As demonstrated, ONRLDB is designed to allow selection of ligands based on various properties and for designing novel ligands or to improve the existing ones.

**Database URL:** http://www.onrldb.org/

## Introduction

Nuclear receptors (NRs) are a superfamily of transcription factors, distinct in their structural organization, whose activity can be switched on and off by small molecules and hormones. Despite major differences in the nature of the ligands to which NRs respond, they share several functional domains and a common structural organization. The N-terminal region has an extremely variable activation function 1 (AF-1) domain that performs activation independent of ligand binding. The central region has a highly conserved DNA-binding domain (DBD); containing two highly conserved zinc fingers, that impart DNA binding specificity, and directs dimerization. The largest domain positioned near the C-terminal is the ligand-binding domain (LBD). The LBD consists of a ligand binding pocket that binds ligand, and an activation function 2 (AF2) region that binds cofactors. The DBD links-up to the LBD by an extremely variable hinge region that contains the nuclear localization sequence and provides flexibility to NRs (1–4). The nuclear receptor (NR) superfamily has two broad categories: endocrine receptors and orphan receptors. Endocrine receptors possess hormones as their endogenous ligands and orphan receptors have no known or universally agreed upon endogenous ligand (5, 6). Endocrine NRs were first described three decades ago as mediators of steroid hormones. Since then, many more members of this superfamily have been characterized; today the list comprises a total of 49 NRs in mouse and 48 NRs in humans (2, 7, 8). The orphan receptors for which endogenous ligands have been identified are referred to as adopted. However, the mere binding of an NR to its ligand does not ensure its adoption until the physiological relevance of the molecule is validated. It is interesting to note that orphan NRs bind to small molecules with lower affinity (Kd values in µM range) unlike endocrine NRs that display high-affinity binding (Kd values in nM range) (5, 9). This difference in affinity is because, orphan NRs possess larger ligand-binding pockets than endocrine NRs. The differences in the affinity might explain the difference in their amenability to pharmacological modulation.

Due to the presence of NRs in diverse cellular processes, they are implicated in the regulation of development, metabolism and inflammation (10–12). Thus, NRs play a significant role in maintaining a normal physiological state (10–12). NRs are one of the protein superfamilies that contain druggable domains and along with G-protein coupled receptors (GPCRs), are on the list of potential drug targets approved by FDA (13). As the endocrine NRs share a large endogenous ligand pool, their use as drug targets may lead to severe side effects. On the other hand, orphan NRs are therapeutically relevant members, especially as they do not share the endocrine NRs endogenous ligands. As a result, many FDA approved drugs, used for the treatment of a broad range of ailments, target orphan NRs. For example, pioglitazone (Actos) and rosiglitazone (Avandia) target PPARγ and are approved for the treatment of type II diabetes; alitretinoin, an agonist for RXR, is approved for the treatment of skin disorders (14). Additionally, recent evidence also suggests that orphan NRs are pertinent in the treatment of other metabolic, autoimmune, inflammatory, and infectious diseases (11, 15–19).

Unfortunately, there is no universal policy to deposit small molecule bioactivity assays before publishing results, unlike sequence, protein structure, and, to some extent, gene expression and mass spectrometry data. The majority of the published data is in unstructured formats such as images, abbreviations and supplementary data; therefore, the data is not searchable. A few public domain bioactive compound resources have been created: ChEMBL, PubChem Bioassay, ChemBank and BindingDB (20–23). These archival databases provide access to millions of compounds and their cognate targets, which are typically from high-throughput screening (HTS) experiments. However, as exemplified in several thematic databases, these mega databases are not efficiently curated (24, 25). Several thematic databases pertaining to NRs biology, or sequence and structure, are available such as NURSA (Nuclear Receptor Signalling Atlas), NURBS (Nuclear Receptor Binding Site Database), NUREBASE, NucleaRDB and NRMD (Nuclear Receptor Mutation Database) and among these databases NURSA and NuclearRDB are maintained (26–30). Over the last two decades, many ligands or related drug molecules targeting orphan NRs have been designed, and their functional relationships have been elucidated. To manage information regarding these small molecules, publically available databases such as NORSE (Novel Nuclear Receptor Super Family Database), IUPHAR-DB (The International Union of Basic and Clinical Pharmacology database), and NRLiSt BDB (Nuclear Receptors Ligands and Structures Benchmarking DataBase) have also been developed (24, 31, 32). These thematic databases, while being informative, unfortunately, are not exhaustively curated and have fewer ligand entries for the orphan NRs family in particular. Additionally, these databases have limited preliminary screening. Therefore, these are deficient in the ability to perform cross searches for cognate ligand–receptor pairing, clustering, and cataloguing tools, etc. that are needed to design poly pharmacological or specific ligands.

In our database, the Orphan Nuclear Receptor Ligand Binding Database (ONRLDB), we took a comprehensive approach to listing experimentally validated ligands with a specific focus on orphan NRs. Our primary aim was to provide ligand structures with all Quantitative Structure Activity Relationship (QSAR) properties, as well as, aligning all quantitative binding measurements from the published experimental data. HTS data such as PubChem Bioassays were excluded to avoid duplications and problems associated with validation of HTS data. We have embedded clustering, cataloguing, and cross searches for ligand-receptor pairs, for data visualization, similarity searching, structural comparisons, prediction of chemical properties, and compound clustering. Compound clustering helps users to eliminate cross-reactive scaffolds and to identify the best scaffolds for a particular NR. These tools are readily accessible, facilitate data mining, aid faster visualization as well as analyse tasks for the design of poly pharmacological and specific ligands against NRs.

## Materials and methods

### Data collection

Data was collected manually from peer-reviewed journals such as the Journal of Biological Chemistry, Nature, Science, and the Journals of the American Chemical Society. Web of Science, Pubmed, and Google Scholar were utilized, and extensive search for all experimental data and structural information was performed for each orphan NR by using trivial names along with the keywords agonist/antagonist/ligands. Search was also performed with synonyms of NRs. Search by NR synonyms that are common with other non-specific proteins was avoided. Ambiguous data and bioassays were excluded. We have manually curated most of the research articles, theoretical reviews, patent reviews and patents as complete as possible. From the review articles, we also curated every reference cited. Details of the NR, ligand structure and function, EC50, IC50, Kd and Ki, etc., were gathered from each article. In certain cases, pEC50 or pIC50 values were manually back-calculated to EC50 or IC50. The structures of the ligands were drawn using Marvin Sketch or Chem Bio Draw Ultra and saved as SDF files. The structures drawn were cross-checked, using PubChem, for accuracy. The comment section provides information on FDA approval status of the ligands and assays performed. Moreover, a list of FDA approved ligands for NRs were mentioned under the ‘Search’ menu.

### Database description

**Figure 1.**
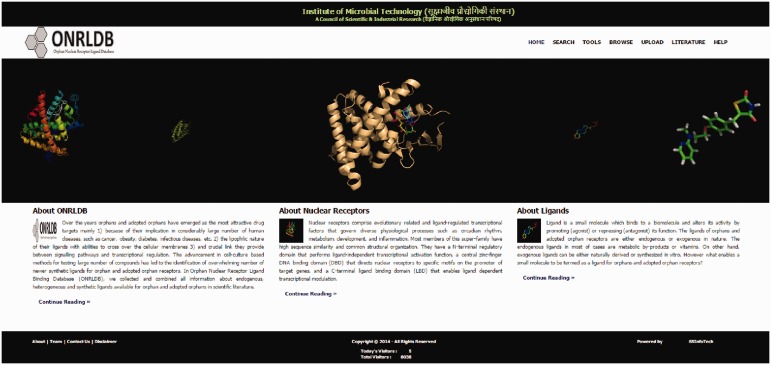
Screenshot of the ONRLDB homepage displaying various functionalities such as search, tools, browse, upload and help.

This database was built using the web server, Apache HTTP, along with the database server, SQL server. Microsoft SQL Server 2008 R2 was used for data storage. The layout of the ONRLDB home page, including the functions: search, tools, browse, upload, literature and help, are shown in Figure 1.

### Structure search

Structure searches can be performed in multiple ways: (i) uploading the SDF file for the molecule, (ii) by entering smiles or (iii) by drawing the 2D structure of the ligand. When a search has to be performed by a structure of compound, the database can be searched via substructure or similarity. All the properties needed for the substructure or similarity search, for example, smiles, etc., were extracted through the Indigo-tool kit of the Bingo server. Smiles are used internally to fire queries on the SQL server to gain the related results. The Bingo utility for the SQL server helps to fire queries; therefore, generating results. Additionally, Jmol was implemented for searching 2D structures from the database.

### Advanced search

The advanced search option can be used to explore ligands using physicochemical properties such as XlogP, molecular weight (MW), the number of rotatable bonds, etc., or by experimental values such as Kd, Ki, IC50 and EC50. This data can be filtered or refined using a specific property or a combination of properties. Each property can be assigned a value for filtering and be restrained by an operator, i.e. equal to, not equal to, greater than or less than. Users can further refine their search by selecting a filter based on NR names or by the function of ligand. The advanced search option is implemented under the search menu.

### Clustering tool

The clustering tool ‘ChemMine' is implemented by using the source code present on the website https://github.com/TylerBackman/chemminetools. Users can perform hierarchical, binning or multidimensional clustering analysis of database compounds using this tool.

### Tree view

To generate the Tree view of the NRs, the tree layout component of d3.js has been implemented. All orphan NRs are represented as the nodes of the tree. By clicking on the nodes, users can view all the structures associated with that particular NR in a slide show. By clicking on the slideshow, users can see a maximized image of the ligands. By clicking on ‘Show all ligands' users can move directly to the ligand page of that particular NR.

### NR catalogue

The NR catalogue is an additional tool to filter compounds and visualize the structures of filtered compounds along with their physicochemical properties. Microsoft Silverlight Pivot Viewer has been used to implement the NR catalogue.

## Results and discussion

### Orphan nuclear receptor ligand binding database (ONRLDB)

The ONRLDB is a comprehensive database of all orphan NRs, their corresponding ligands and small molecule modulators that encompass a broad pharmacological profile. All the 34 members of the orphan NR family and their corresponding experimentally verified ligands were covered. This exercise yields ∼11 000 small molecule modulators of which 6390 are unique entries. Several of the other small molecule modulators that have been curated share scaffolds and are crossover ligands. This database is aimed at specific and poly pharmacological ligand discovery for this category of therapeutic targets. Various tools are included to facilitate this objective, e.g. advanced structure search, clustering, cataloguing and tree presentation of cognate ligand–receptor pairs. The ONRLDB is accessible via a simple, user-friendly interface at www.onrldb.org (Figure 1). The interface links to home, search, tools, browse, upload, literature and help menus.

### Data retrieval/search from ONRLDB

**Figure 2.**
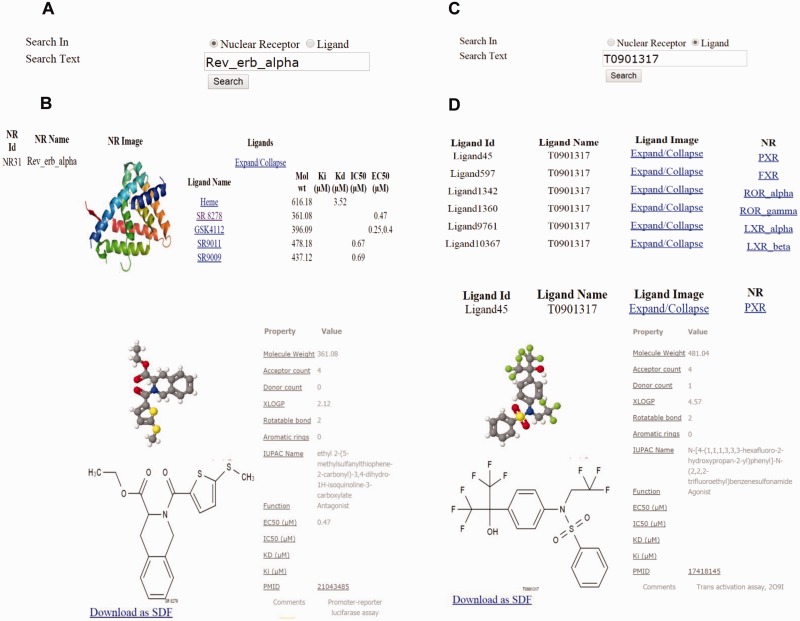
Examples of text-based and property based advanced search in ONRLDB. (**a,c**) Protein and ligand-based text search. (**b**) Results of the NR text search using Rev-erb alpha as an example; the ligands of the particular receptor are displayed. (**d**) Results of the ligand text search using T0901317, a common ligand for PXR, FXR, LXR and RORs, which displays all the cognate receptors.

**Figure 3.**
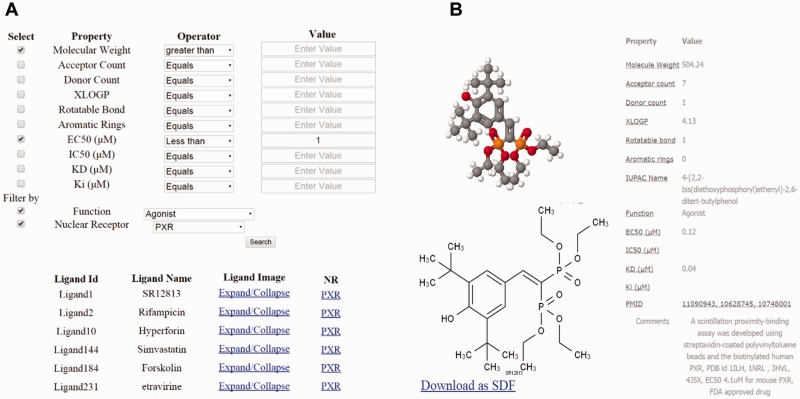
(**a**) Dialog box of the property based advanced search. (**b**) Results of the property based advanced search using parameters such as molecular weight (MW) of 400 Daltons and above, EC50 <1, and an agonist of PXR.

**Figure 4.**
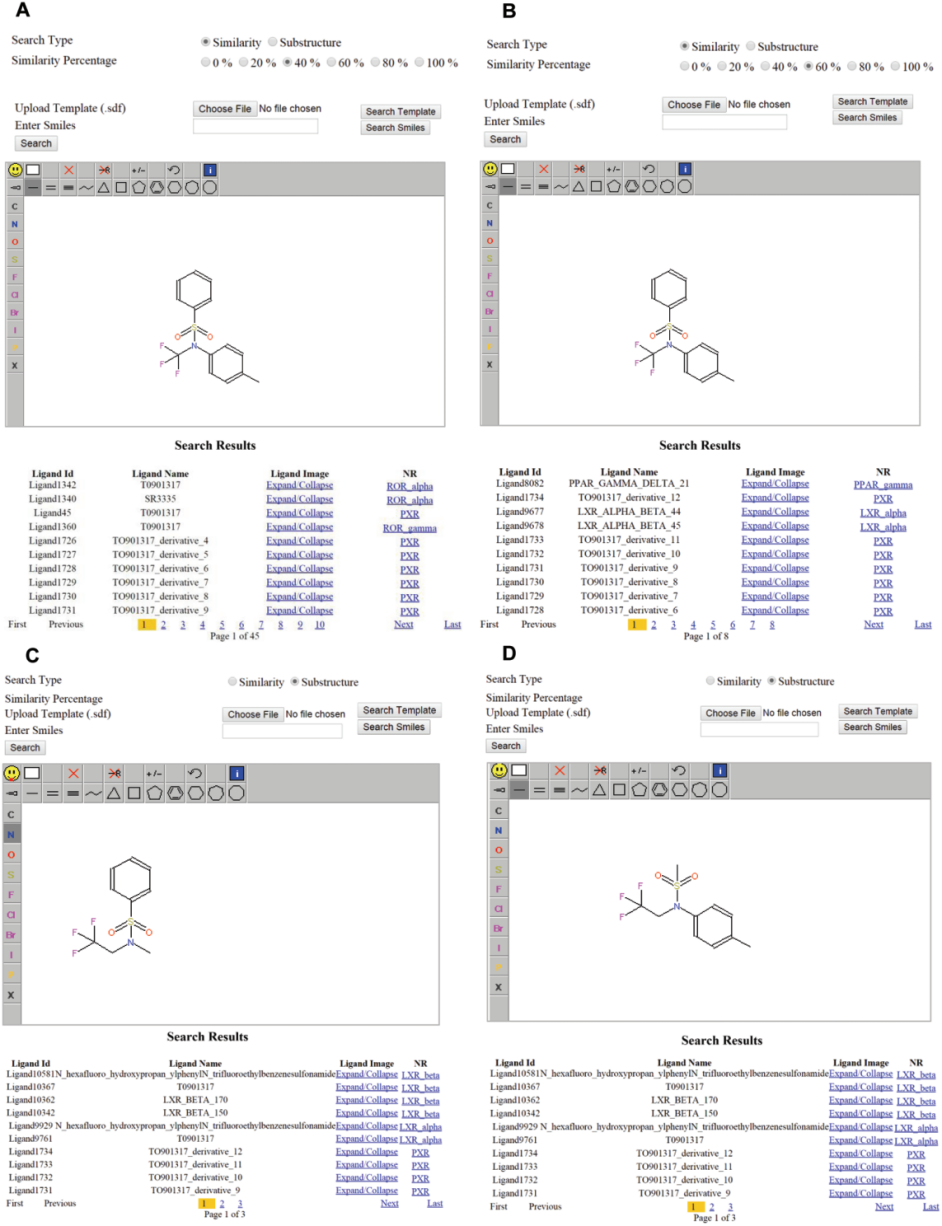
Examples of substructure and similarity-based structure search. (**a**) Similarity search for the same ligand at 40% and (**b**) 60% and the result. (**c**) Substructure search using one-half of the ligand T0901317 and the result. (**d**) Substructure search using the second half of the T0901317 and the result.

The interface consists of different search options such as text search for both NR and ligand (Figure 2a-d), advanced search (Figure 3a and b), and structure search (Figure 4). A specific NR search will provide an output, as available and curated, of the NR ID, a PDB image and an expandable list of all cognate ligands with their corresponding physicochemical and experimental properties. A particular ligand search will provide an output of the ligand image, its structure, the IUPAC name and references. Additionally, the search results will include manually curated experimental information such as Kd, Ki, IC50, EC50 and physicochemical properties such as the number of aromatic rings, the number of rotatable bonds, XlogP, hydrogen donor and acceptor count and MW. Specific ligand search also provides information on one or more cognate NRs, and this search function will be helpful in identifying crossover, multi-target ligands. Users can directly expand on any cognate ligand or cognate NR searched via the above options. Using the advanced search, users can be more precise in selecting ligands, based on their physicochemical or experimental properties mentioned previously. Any specific or combinations of properties can be selected and regulated, via filter using an operator (equals to, not equal to, less than and greater than) and value. This search can be further narrowed down by selecting a specific NR and/or ligand function type such as an agonist, antagonist, and inverse agonist. This exercise will list all ligands, properties and cognate NRs. The ligand interface also allows downloading of the ligand 2D structure.

### An online tool for ligand structure search

Structure searches can be performed by substructure or by similarity percentage. To search ligands by structure, users can draw the partial or complete structure, using a 2D structure drawing interface, and retrieve similar compounds. Powerful editors are embedded within the structure page. Users can also search for similar ligands by entering the smiles format of the particular ligand or by uploading the SDF file of the ligand. The ligand page includes information about its function, target, experimental, and QSAR properties. This page also displays the 2D structure that can be downloaded. In the substructure search, users can draw the structure of the query on a Jmol java applet that runs on the server side. The server script stores this information into a temporary file. A .NET script is written, and an Indigo (Bingo) tool is used to search the query molecule throughout the database for structurally similar molecules.

### An online tool for presentation of cognate ligand–receptor pairs as a tree

**Figure 5.**
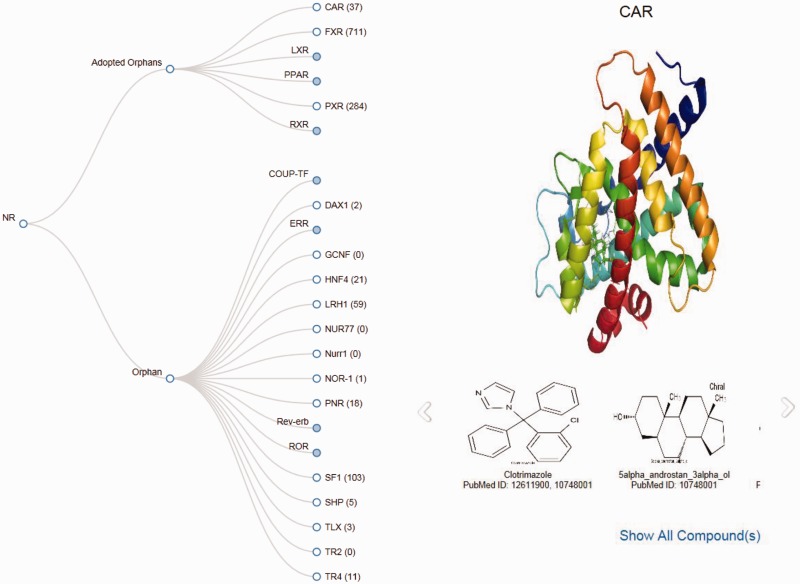
Screenshot of the Tree page displaying images of the structure of CAR and its ligands.

This tool provides a Tree representation of orphan or adopted orphan NRs, and provides the corresponding number of ligands of all NRs in a single user-friendly window. Any of the NRs can be selected, and the structure and cognate ligands can be viewed in a single window with a link to the view the ‘all ligands’ page (Figure 5).

### Online tools for clustering analysis

**Figure 6.**
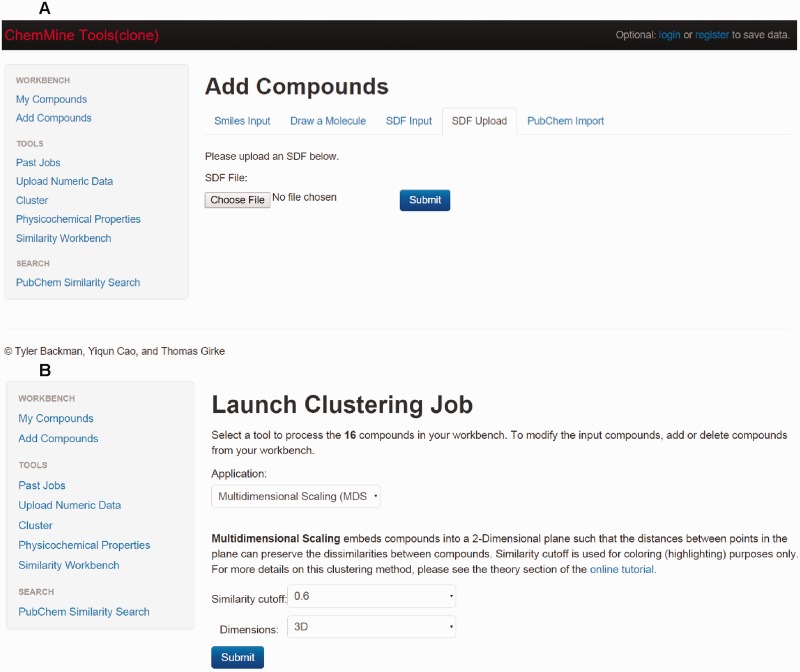
Screenshot of ChemMine tools (clone). (**a**) Screenshot of ChemMine tool displaying the compound page. (**b**) Screenshot of ChemMine tool displaying the clustering page.

Clustering is a method that identifies or classifies drugs based on their structural backbone or scaffolds. Clustering is a crucial method for structural biologists and chemists in the process of drug discovery. ChemMine is a tool for small molecule data analysis. Using ChemMine tools (clone), users can visualize the data as well as perform the structural comparison, similarity searches, and clustering analysis (Figure 6a and b). Included are three types of clustering analysis: hierarchical clustering, multidimensional scaling (MDS), and binning clustering (33).

### An online tool for cataloguing: NR catalogue

**Figure 7.**
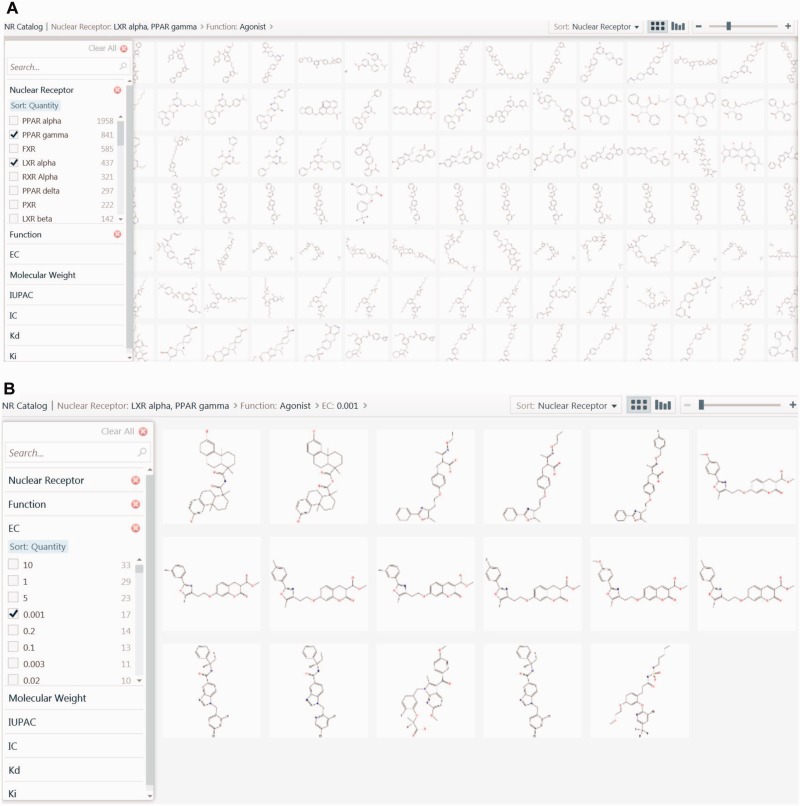
Screenshot of the NR catalogue page. (**a**) The screenshot is showing images of common ligands for PPARγ and LXRα. (**b**) The screenshot is showing best ligands of PPARγ and LXRα, which have been selected based on their agonistic property and extremely significant EC50 value of 0.001 μM.

The NR catalogue is another user-friendly tool designed to filter and visualize compounds based on the NR, the ligand function, MW, and experimental values such as Kd, Ki, IC50, EC50. Structures of all ligands were embedded in the NR catalogue to allow users to visualize and compare the structures of ligands belonging to different NRs. Ligands from one or more NR can be selected and filtered on the basis of function, MW and experimental values. This tool is valuable in segregating, cataloguing and visualizing ligands, on the basis of their function and their biological potency. Additionally, this tool is useful to identify scaffolds that may contribute to better receptor binding. The capability of the NR catalogue for visualizing ligands of NRs with similar biological function, together with clustering, in identifying common scaffolds, will be extremely beneficial in poly pharmacological drug design (Figure 7a and b).

### Model exercises for identifying unique or common scaffolds aimed at specific or poly pharmacological ligand discovery

**PPARγ and LXRα are in the crosshairs of inflammation and infection.** PPARγ (Peroxisome proliferator-activated receptor γ) is one of the orphan NR that has been adopted and is well known for its role in the regulation of glucose and lipid metabolism. PPARγ maintains glucose homeostasis by increasing the expression of GLUT4 and other factors, which increases the insulin sensitivity (34). Rosiglitazone and pioglitazone (PPARγ agonists) have been used in anti-diabetic compositions because of their ability to improve insulin sensitivity without affecting the secretion of insulin from beta cells (35). Unfortunately, PPARγ agonists have various adverse side effects. Despite this, new structural classes of full and partial agonists against PPARγ are still in demand. PPARγ activation promotes anti-inflammatory phenotypes, by inducing arginase-1 expression (a key marker of alternative macrophage phenotype) and IL4 (Interleukin 4) secretion (35). PPARγ blocks the induction of RORγt (RAR-related orphan receptor γt) in response to TGFβ (Transforming growth factor β) and IL6 (Interleukin 6) and inhibits Th17 driven inflammatory diseases, e.g. EAE, inflammatory bowel disease (IBD) and collagen-induced arthritis (CIA) (36).

Liver X receptors (LXRs) play a significant role in glucose metabolism and homeostasis. In the fed state, oxysterols activate LXRs, inhibiting the gluconeogenesis and promoting glycogen synthesis by upregulating the expression of glucokinase (35). As well as, LXRs are crucial for cholesterol metabolism. Oxysterols generated by cholesterol degradation activate LXRs in the liver, which promote the formation of bile acids and avoids the accumulation of cholesterol (34). LXRs also induce *de novo* lipogenesis and increase circulating triglycerides. LXRs regulate genes involved in reverse cholesterol transport, bile acid metabolism, and intestinal cholesterol absorption (35). These processes have been reported to be anti-atherogenic, and LXR activation has been shown to protect mice against the development of atherosclerosis. In addition to its role in metabolism, LXRs have also been reported to regulate the expression of several inflammatory genes in macrophages. For example, via suppression of the inflammatory genes COX-2, MMP-9 and iNOS, with the induction of the arginase-2 gene, and additional inhibition of NF-kB, LXRα activation promotes alternative macrophages (35). Furthermore, activation of LXRα has also been reported to inhibit Th17 cell differentiation, and the progression of autoimmune diseases such as experimental autoimmune encephalitis (EAE) (36).

Consequently, PPARγ and LXRα agonists can act as potential drugs to treat several metabolic syndromes. Given their anti-inflammatory nature, the design of a poly pharmacological agonist that can target both receptors would be more effective to treat inflammatory diseases such as allergies, autoimmune diseases, systemic diseases and atherosclerosis. However, unlike the convergence of PPARγ and LXRα in the inflammatory condition, there is a dissonance in the case of *Mycobacterium tuberculosis* (*M. tuberculosis*) infection. Recent studies on PPARγ from our group revealed that PPARγ allows *M. tuberculosis* to survive inside macrophages by inducing foamy biogenesis and IL10 (Interleukin 10) expression (17). Whereas, despite its anti-inflammatory role, LXRα has been shown to inhibit *M*. *tuberculosis* survival in human macrophages by different mechanisms (17). Hence, to treat *M*. *tuberculosis* it is important to design agonists, which consist of scaffolds specific for LXRα that are not common for PPARγ.

**Figure 8.**
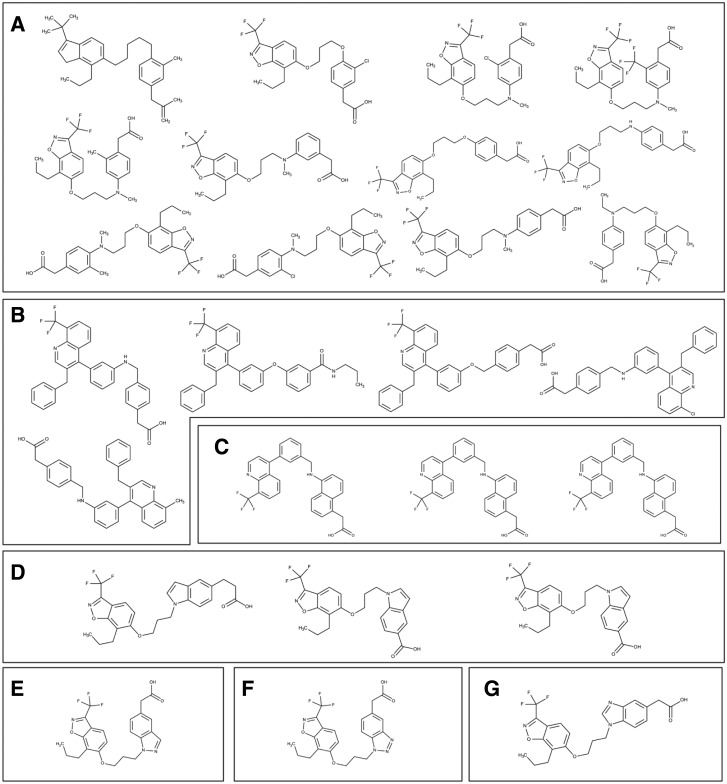
Structures of the common scaffolds in LXRα and PPARγ.

**Figure 9.**
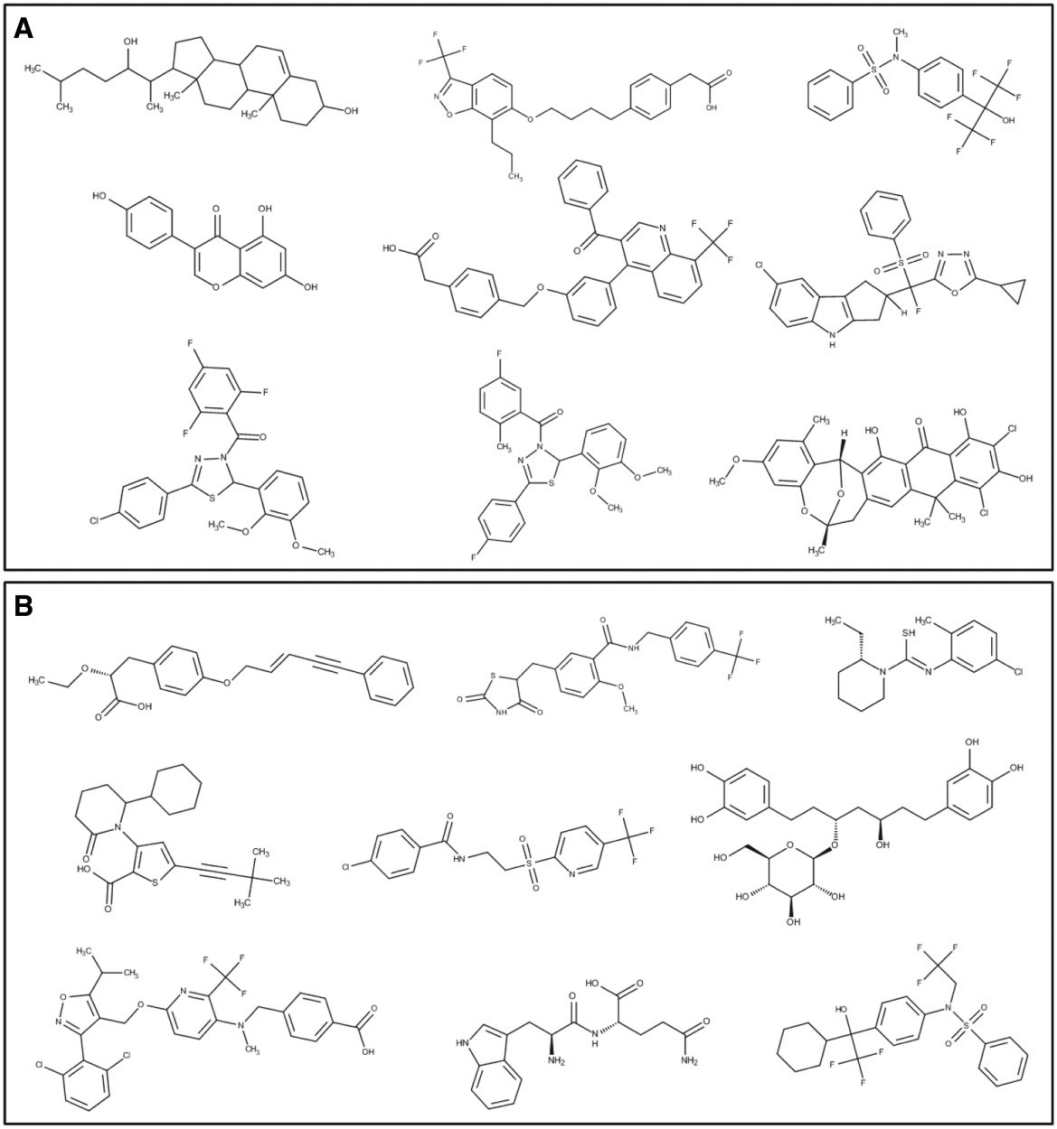
Structures of the unique scaffolds in (**a**) LXRα and (**b**) PPARγ, with a few representative examples of various bin clusters.

To reveal the unique and overlapping structures, we compared different small molecules targeting LXRα and PPARγ using the OpenBabel program (37). Upon comparison of LXRα and PPARγ, we observed 26 structures in common, 491 unique to LXRα and 3337 unique to PPARγ. We found seven scaffolds among the 26 common structures; viz benzoisoxazole (12 structures), quinoline (5 structures), quinolinyl benzyl napthalene-1-amine (3 structures), indolyl-propoxybenzoisoxazole (3 structures), indazolyl-propoxyl-benzoisoxazole (1 structure), benzo-triazolyl-propoxy-benzoisoxazole (1 structure) and benzoimidazolyl-propoxy-benzoisoxazole (1 structure) (Figure 8). Clustering analysis was performed by using ChemMine tool and the steps involved in clustering methodology are: dataset generation, correctly selecting the similarity measurement, clustering of the dataset, analysis of the data, and, eventually, visualization of the clustered data (38, 39). Binning clustering was performed to explore the scaffolds among the unique molecules of LXRα and PPARγ using a similarity cut-off (Tanimoto coefficient) of 0.4. Binning partitions of LXRα and PPARγ resulted in 13 and 74 clusters respectively and provided a varied number of molecules. LXRα was found to have a broad range of scaffolds; tetradecahydro cyclopentaphenathrene, quinoline, diphenyl thiohydroxylamine, benzyl diphenyldihydroxythiadiazole (observed in two clusters), chromene, benzoisoxazole, tetrahydrocyclopentaindole and dioxahexacyclo pentacosanonaene (Figure 9a). On exploring the scaffold diversity of PPARγ, we detected, phenoxy pentenyl benzene, benzyl thiazolidinyl methyl phenyl methanamine, benzyl pyridinylthioethanamine, propanamido carbamoyl butanoic acid, piperidinyl methylene analine, cyclohexyl thiophenyl piperidine, benzyl phenylisoxazolyl methoxy pyridinamine, diphenylheptanyl-oxy-tetrahydropyran and cyclohexyl methyl phenyl thiohydroxylamine (Figure 9b). We expect that clusters represented by one or two molecular scaffolds can be populated using medicinal chemistry and structure–activity relationships (SAR) studies; thus leading to the development of small molecules targeting specifically to LXRα or commonly to both LXRα and PPARγ. This critical analysis surveys an essentially limited range of currently uncharacteristic scaffolds, primarily drawn from the literature, that have found applications in medicinal chemistry; some being therapeutically potent and others that are already in clinical use.

**CAR and PXR: the xenobiotic cleansers.** The promiscuous adopted orphan NRs, CAR (Constitutive Androstane Receptor) and PXR (Pregnane X Receptor), sense a wide variety of structurally diverse compounds. CAR and PXR can detect toxic exogenous chemicals and by-products of endogenous metabolic compounds, and facilitate their elimination (40). CAR principally modulates metabolic detoxification via regulation of CYP2B (Cytochrome P450 2B6) genes and transferases in hepatocytes, as well as transporters such as OATP2 (Organic Anion Transporting Polypeptide 2), leading to a net clearance of xenobiotics from the blood (40, 41). Whereas, metabolic detoxification is predominantly modulated by PXR, through CYP3A (Cytochrome P450 Family 3 Subfamily A) isoforms, as well as other mechanisms of xenobiotic metabolism. For example, through carboxylesterases, alcohol dehydrogenases, and transporters, i.e. OATP2 and MRPs (Multidrug Resistance-associated Proteins). CAR and PXR also regulate the transporters involved in bile acid elimination (40). Despite binding to structurally different ligands, these two NRs share common ligands and target genes that have similar response elements. Common ligands to CAR and PXR include ethinyl estradiol, diethylhexyl phthalate and clotrimazole. Even though these receptors share some common ligands, the structural analysis confirms that CAR is less promiscuous because of its smaller binding pocket (40, 41). Hence, PXR and CAR may play complementary roles in sensing harmful compounds inside the body. Apart from their role in drug metabolism and detoxification, both PXR and CAR were reported to modulate negatively gluconeogenesis and diabetes (42). Hence, while addressing drug metabolism or a metabolic disease like diabetes, designing poly pharmacological drugs that target both PXR and CAR would be of a better strategy. PXR has also been described to inhibit inflammation by strongly inhibiting NF-kB-mediated inflammatory pathways while CAR does not possess any well-reported role in inflammation (42). Hence, to treat inflammatory diseases, it is important to design agonists, which consist of specific scaffolds for PXR only.

**Figure 10.**
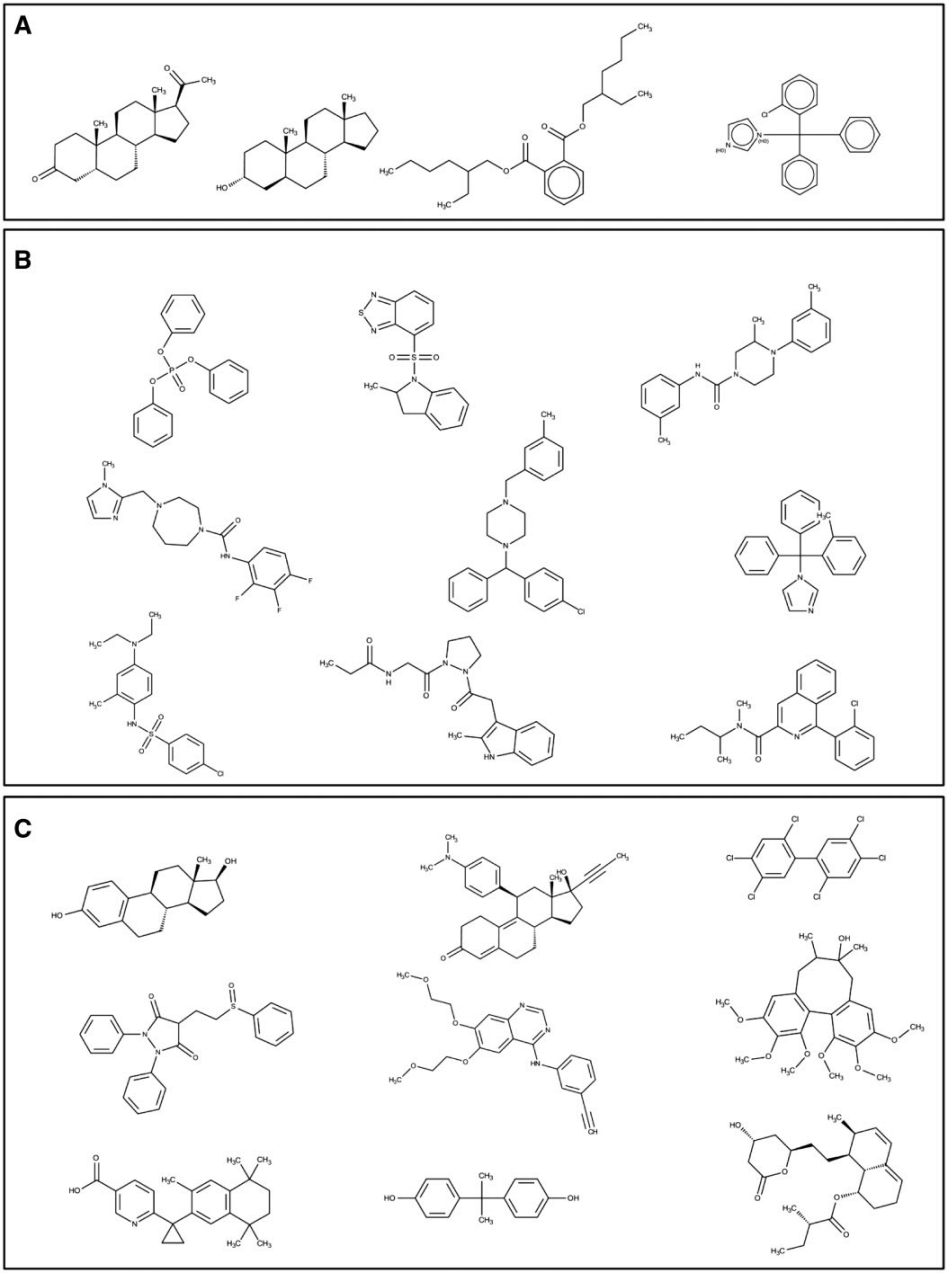
Structures of (**a**) common and (**b**, **c**) unique scaffolds in CAR (b) and PXR (c), with a few representative examples of various bin clusters.

PXR and CAR’s unique and overlapping structures were compared; we found four structures in common, 34 unique to CAR, and 240 unique to PXR. The four common structures were considered to comprise three different scaffolds: hexadecahydro cyclopentaphenathrene, trityimidazole, and ethylphenyl benzene dicarboxylate (Figure 10a). Binning clustering of CAR and PXR (as described above) resulted in 24 and 76 clusters respectively. On exploring scaffolds diversity of CAR ligands, we detected piperazinediphenylthiohydroxyamine, diphenyl-tolyl-methylimidazole, imidazolyl-methyldiazepanyl methyl aniline, isoquinoline, pyrozolidinyl propanamide, cyclohexadienyl piperazinyl methyl analine, benzylchlorophenyl phenylmethyl, methyl indolinyl-thiobenzothiadiazole and triphenyl phosphate (Figure 10b). While decahydro cyclopentaphenathrene, dodecahydro cyclopentaphenathrene, trichlorophenyl benzene, diphenylthioethyl pyrazolidine, tricyclohexadecahexene, quinazoline, tetrahydronapthalene, diphenylmethane and hexahydronapthalene were unique scaffolds to PXR (Figure 10c). We postulate that poly pharmacological drug design that accommodates both the common and unique scaffolds of CAR and PXR would be desirable, as they would likely bind better and have more biological potency. The common scaffolds of PXR and CAR represented in Figure 10a can be used as starting point for the discovery of poly pharmacological ligand to target drug metabolism or metabolic diseases like diabetes. Whereas PXR unique scaffolds displayed in Figure 10c can be used as starting point for the discovery pharmacological ligand to treat inflammatory diseases like inflammatory bowel disease (IBD).

## Significance of the database

In ONRLDB, we have accumulated all information on experimentally validated ligands from the literature. PubChem Bioassays were not included in ONRLDB, to avoid problems associated with HTS data. We reviewed [mt]2000 articles for all relevant information, regarding all 34 orphan NRs and their ligands, to compile ONRLDB. The ONRLDB consists of a total of 10683 ligands, in which 10132 ligands bind orphans NRs that are adopted, and the remaining 551 ligands are specific for the remaining 23 orphan NRs. ONRLDB not only lists all the orphan NRs, but it also includes ligands with a wide array of pharmacological profiles. Furthermore, though most of the orphan NRs share structural similarity, they may not share similar biology or cognate ligands. To help users compare, analyse and identify scaffold similarity we have implemented structure, similarity, clustering, and cataloguing tools into the ONRLDB. This database will help users to design specific ligands and to avoid cross-talk between receptors, by identifying unique scaffolds. These functions also assist in the design of poly pharmacological ligands, which target different NRs that have similar biological functions.

Compared with other databases, ONRLDB has a clear edge in drug design for orphan NRs targets. NRLiSt BDB is focused only on those NRs with at least one experimental structure available; consequently, covering only 27 of the 48 total human NRs, and 15 of the 34 orphan NRs. For the 15 orphan NRs covered in NRLiSt BDB, it includes of 5945 ligands only. NRLiSt BDB also lacks ligands with alternative pharmacological profiles. Additionally, orphans NRs such as TR4, LRH1, SHP, PNR, HNF-4α, ERRγ, RORβ, Rev-Erbβ and DAX1 were not covered. Clearly, ONRLDB provides more extensive coverage than NRLiSt, for both, the number of ligands and the number of orphan NRs covered. ChEMBL covers all the 34 orphan NRs. Among orphan NRs BindingDB has no entries for TLX, SHP and Rev-erbβ, even though, their ligands are reported in the literature. Furthermore, data included in ChEMBL and BindingDB may not applicable for direct use in stringent drug discovery approaches due to the inclusion of HTS data. Besides this, there are several errors such as data duplication, improper citations and inclusions of unrelated assays for NRs. Hence, before using this data for further analysis such as the identification of specific scaffolds, users have to first manually curate the data to avoid duplicates, HTS data, and improper data, which is not ideal for many users. IUPHAR-DB mainly consists of information about G protein-coupled receptors, voltage/ion gated channels, and NRs. This database has a ligand count for orphan NRs of ∼210 ligands, and the data it provides regarding agonists and antagonists is not sufficient for present day drug discovery requirements.

Databases that lack manual curation, unfortunately, have errors. For example, ChEMBL and BindingDB consist of 22 and 7 ligand entries for TR4 (Testicular Receptor 4) respectively; however, many of these entries are inappropriate as TR4 has a synonym TAK1 that is a name shared with a protein kinase (TAK1) of the MLK family. In ONRLDB, we have avoided such issues with synonyms and implemented a drop down menu for all NRs from where the user can easily select the required NR.

## Conclusion and future prospects

ONRLDB includes manually curated orphan NR ligands and is as complete as possible. Through an interactive platform and tools, ONRLDB facilitates cognate ligand-orphan NR searches, structure and similarity searches, clustering and cataloguing, to aid in the discovery of unique and common scaffolds. This publicly available database can be browsed by NR name, ligand name, ligand structure, and fingerprint-based chemical similarity search; additionally ONRLDB can be navigated through a video tutorial under the help menu. ONRLDB will be extremely helpful in the design and discovery of small molecule ligands for orphan NRs, which are known to modulate important pathological processes such as cancer, autoimmune, neurodegenerative, and cardiovascular disorders. In future versions of ONRLDB, we plan to include several tools such as automated docking, principal component analysis, and the functional downstream interactomes of the ligand–receptor pair. Orphans NRs are promising therapeutic targets, and the data is expected to increase exponentially. Therefore the data will be subjected to yearly review, and ONRLDB will be updated to reflect advances in the field. We also welcome suggestions and critical inputs through the email link that has been provided on the upload page.
